# GABAergic Inhibitory Interneuron Deficits in Alzheimer’s Disease: Implications for Treatment

**DOI:** 10.3389/fnins.2020.00660

**Published:** 2020-06-30

**Authors:** Yilan Xu, Manna Zhao, Yuying Han, Heng Zhang

**Affiliations:** Neurodegeneration and Neuroregeneration Laboratory, Department of Basic Medicine, School of Medicine, Shaoxing University, Shaoxing, China

**Keywords:** GABA inhibitory interneurons, cognitive deficits, Alzheimer’s disease, amyloid β-protein, PV inhibitory interneurons

## Abstract

Alzheimer’s disease (AD) is a neurodegenerative disorder characterized clinically by severe cognitive deficits and pathologically by amyloid plaques, neuronal loss, and neurofibrillary tangles. Abnormal amyloid β-protein (Aβ) deposition in the brain is often thought of as a major initiating factor in AD neuropathology. However, gamma-aminobutyric acid (GABA) inhibitory interneurons are resistant to Aβ deposition, and Aβ decreases synaptic glutamatergic transmission to decrease neural network activity. Furthermore, there is now evidence suggesting that neural network activity is aberrantly increased in AD patients and animal models due to functional deficits in and decreased activity of GABA inhibitory interneurons, contributing to cognitive deficits. Here we describe the roles played by excitatory neurons and GABA inhibitory interneurons in Aβ-induced cognitive deficits and how altered GABA interneurons regulate AD neuropathology. We also comprehensively review recent studies on how GABA interneurons and GABA receptors can be exploited for therapeutic benefit. GABA interneurons are an emerging therapeutic target in AD, with further clinical trials urgently warranted.

## Introduction

Alzheimer’s disease (AD) is the most common neurodegenerative disease. AD is characterized clinically by severe cognitive deficits and pathologically by amyloid plaques, neuronal loss, and neurofibrillary tangles (Selkoe, 1999; Selkoe and Hardy, 2016; Wyss-Coray, 2016; Polanco et al., 2018). AD patients often have learning deficits, memory loss, language difficulties, confusion in place and time, and emotional and other behavioral changes (Selkoe and Hardy, 2016; Polanco et al., 2018). These changes are not only a burden to the individual but also to families, communities, and healthcare systems (Alzheimer’s Association, 2019). However, the molecular mechanisms underlying the cognitive deficits seen in AD are unclear, and there remains no effective treatment to slow or halt progression of AD.

Many factors are known to contribute to AD pathophysiology (Mucke et al., 2000; Mucke, 2009; Mucke and Selkoe, 2012), with abnormal aggregation of amyloid β-protein (Aβ) in the brain a likely dominant initiating factor (Huang and Mucke, 2012; Barage and Sonawane, 2015; Musiek and Holtzman, 2015; Wiseman et al., 2015; Donohue et al., 2017). Aβ is formed from cleavage of amyloid precursor protein (APP) by beta (β)-(BACE1) and gamma (γ)-secretase, and Aβ exists in different forms including monomers, oligomers, fibrils, and senile plaques (Whitehouse et al., 1982; Tanzi and Bertram, 2005; Canter et al., 2016; Riek and Eisenberg, 2016). Excessive Aβ can affect the expression of synapse-related proteins, decrease dendritic spine density, inhibit excitatory synaptic transmission, affect synaptic plasticity, and cause cognitive impairments both in AD patients (Donohue et al., 2017; Evered et al., 2019; Wen et al., 2019) and AD mouse models (Selkoe, 2019; Zott et al., 2019) including Tg2576, 5xFAD, 3xTg-AD, APP/PS1, hAPP-J20, and hAPPJ9/FYN mice (Verret et al., 2012; Flanigan et al., 2014; Palop and Mucke, 2016; Shu et al., 2016; Cattaud et al., 2018; Lerdkrai et al., 2018). A novel *App*^*NL–F/NL–F*^ knock-in AD model mouse shows age-dependent accumulation of Aβ, neuroinflammation, and neurodegeneration similar to AD patients (Wang et al., 2017c; Shah et al., 2018). Recent studies performed in this model indicate that, in the early stages of AD, synaptic dysfunction originates in the lateral entorhinal cortex (LEC) and then spreads to other brain areas including the hippocampus in combination with synaptic hyperexcitation, severely disrupting excitatory–inhibitory inputs and resulting in synaptic imbalance and dysfunctional synaptic homeostasis (Petrache et al., 2019; Shi et al., 2019). Dysfunctional synaptic homeostasis induces a decrease in long-term potentiation (LTP), and it has been shown that high Aβ concentrations inhibit LTP, which is essential for the normal development of cognitive functions such as learning and memory, whilst enhancing long-term depression (LTD) (Wang et al., 2004; Shankar et al., 2007; Li G. et al., 2009; Tackenberg and Brandt, 2009; Mucke and Selkoe, 2012). LTP inhibition and LTD enhancement disrupt normal neural network activity and may induce cognitive impairment in AD (Palop and Mucke, 2010). Network activities supporting cognition, including activation and deactivation deficits, abnormal oscillatory rhythmic activity, and network hypersynchrony, are altered in AD (Sperling et al., 2009), schizophrenia (Seidman et al., 2014), epilepsy (Fahoum et al., 2013; Oser et al., 2014), and other neurological and psychiatric diseases (Anticevic et al., 2012). It has been shown that network hypersynchrony and altered oscillatory rhythmic activity may lead to cognitive abnormalities in AD (Palop and Mucke, 2016). During normal brain activity, excitatory and inhibitory neurons are co-activated and maintain normal network activities (Iaccarino et al., 2016). The balance between neuronal excitation and inhibition is disrupted in neurodegenerative and psychiatric disorders including epilepsy, schizophrenia, Parkinson’s disease (PD), and AD (Shetty and Turner, 2000; Marin, 2012; Southwell et al., 2014; Tyson and Anderson, 2014). γ-aminobutyric acid (GABA) inhibitory interneurons (GABAergic neurons) regulate excitatory neurons in different brain regions through GABA release. Imbalanced GABAergic and glutamatergic transmission has been shown to impair adult neurogenesis in animal models of AD (Sun et al., 2009). Thus, abnormally increased neural network activity in AD may be due to excitatory-inhibitory imbalance.

A recent clinical imaging study combining positron emission tomography with a novel analytical framework showed that distant Aβ induces regional metabolic vulnerability, and the interaction between local Aβ and a vulnerable environment drives the clinical progression of dementia (Pascoal et al., 2019). Similar results were obtained in transgenic Aβ rats that do not form neurofibrillary tangles, suggesting a novel mechanism of cognitive deterioration (Pascoal et al., 2019). Therefore, the causal link between Aβ and AD remains controversial, and further work is still required to fully establish how Aβ participates in the definition, etiology, and diagnosis of AD. The genetic risk factors for AD may act through both Aβ-dependent and Aβ−independent mechanisms to influence disease onset and progression (Morris et al., 2014, 2018; van der Kant et al., 2020). Despite this, the presence of extracellular Aβ deposition as neuritic plaques and intracellular accumulation of hyperphosphorylated Tau (pTau) as neurofibrillary tangles remain the primary neuropathological diagnostic criteria for AD (Long and Holtzman, 2019). Moreover, plasma Aβ is a reliable biomarker for AD in the clinical setting, along with other biomarkers (Wang et al., 2019). Aβ still plays an important role in AD pathology, and here we explore both Aβ−dependent and Aβ−independent mechanisms of neurocognitive decline and how they might be exploited for clinical benefit.

## Studies on Excitatory Neurons in AD

Initial studies on the pathological changes and cognitive impairment induced by Aβ focused on Aβ’s effect on excitatory neurons. In excitatory neurons, Aβ can reduce synaptic transmission by activating the glutamate N-methyl-D-aspartate receptor (NMDAR) (Liu et al., 2004; Kullmann and Lamsa, 2007; Sinnen et al., 2016; Wang and Reddy, 2017). Aβ inhibits excitatory synaptic transmission, reducing neuronal network excitability (Wang et al., 2004; Shankar et al., 2007; Li S. et al., 2009; Tackenberg and Brandt, 2009; Mucke and Selkoe, 2012). However, many studies have shown that Aβ can also induce aberrant neural network activity (Verret et al., 2012; Palop and Mucke, 2016). Aβ leads to rapid excitatory amino acid transporter (EAATs) mislocalization and internalization and alters the clearance of glutamate, resulting in excitatory neurotoxicity during AD development (Scimemi et al., 2013). Synaptic damage can be significantly inhibited by treatment with the vitamin E derivative, Trolox (Scimemi et al., 2013). An intrasynaptic vesicle APP domain also promoted the release of the excitatory neurotransmitter glutamate (Yao et al., 2019). Recent studies in APP/PS1 mice have shown that, in the early stages of AD, microRNA-34a expression increases before Aβ production and cognitive impairment (Xu et al., 2018). MicroRNA-34a caused synaptic damage and cognitive impairment by inhibiting NMDAR and α-amino-3-hydroxy-5-methyl-4-isoxazolepropionic acid (AMPA) receptor expression (Xu et al., 2018). Moreover, increased microRNA-34a expression inhibited synaptogenesis by suppressing the expression of two synaptic proteins, synaptotagmin I and syntaxin 1A, in AD patients (Agostini et al., 2011; Sarkar et al., 2016). microRNA-34a knockdown *in vitro* significantly increased NMDA and AMPA receptor expression, thereby inhibiting synaptic damage and cognitive deficits (Agostini et al., 2011; Sarkar et al., 2016).

AD pathogenesis is also associated with significant cholinergic and glutamatergic neurotransmitter system dysfunction, including changes in the levels of these neurotransmitters and neuronal network dysfunction in AD patients (Rossor et al., 1981; Dickson and Murray, 2015; Ferreira-Vieira et al., 2016; Kamkwalala and Newhouse, 2017; Mesulam et al., 2019). Furthermore, the basal forebrain cholinergic system (BFCS) undergoes severe atrophy in AD patients (Grothe et al., 2012) as well as in AD animals (Salehi et al., 2006; Burke et al., 2013; Zhao et al., 2017). Based on these studies, US Food and Drug Administration (FDA)-approved drugs for AD can be divided into two categories: the acetylcholine inhibitors including tacrine, donepezil, galanthamine, and rivastigmine, which enhance residual cholinergic activity to treat mild AD, and memantine, an NMDAR antagonist (Santos et al., 2016; Michalska et al., 2017; Rojas-Gutierrez et al., 2017; Behrens et al., 2018; Canevelli et al., 2018; Ettcheto et al., 2018; Khoury et al., 2018; Sahoo et al., 2018). However, these therapeutic drugs do not fundamentally delay disease progression and are required at large doses that induce adverse reactions; they are also too expensive for many patients (Alzheimer’s Association, 2019). Therefore, there is a clinical imperative to develop different treatment schemes for different stages of AD development (Graham et al., 2017). Regulating the expression of NMDA and AMPA receptors using NMDAR antagonists to inhibit excitatory neurons and EAAT expression may improve synaptic damage and cognitive deficits, but there is clearly a need for further studies on regulating excitatory neurons and the cholinergic system to identify new therapeutic modalities.

## The Role of γ-Aminobutyric Acid Inhibitory Interneurons in AD Pathogenesis

Studies have demonstrated that GABA is an important inhibitory neurotransmitter that balances neural excitability and inhibition and is ubiquitously expressed in the brains of mammals including humans (Isaacson and Scanziani, 2011; Bown and Shelp, 2016; Chen et al., 2017; Tang, 2019). GABA’s inhibitory function is mainly mediated by three different GABA receptors: GABA_*A*_, GABA_*B*_, and GABA_*C*_ (Li et al., 2016). GABA inhibitory activities can be divided into two main types: phasic inhibition and tonic inhibition. In phasic inhibition, during the action potential, the membrane depolarizes and GABA is released from presynaptic vesicles to rapidly increase GABA concentrations in the synaptic cleft. GABA activates GABA receptors in the postsynaptic membrane, decreasing postsynaptic neuron excitability (Farrant and Nusser, 2005). Phasic inhibition is induced by the transient or phasic activation of GABA receptors by GABA from presynaptic vesicles (Farrant and Nusser, 2005; Li et al., 2016). The postsynaptic γ2 subunits of GABA_*A*_ receptors have been shown to be the primary mediators of phasic inhibition (Schweizer et al., 2003; Farrant and Nusser, 2005). Extrasynaptic GABA_*A*_ receptors containing the pi (π) subunit mediate tonic inhibition in most brain regions, and the alpha (α)-5 and delta (δ) subunits are the major GABA_*A*_ receptors mediating inhibition in the hippocampus (Glykys et al., 2008). Hippocampal neurons receive inhibitory charges from tonic inhibition that account for ~75% of the total inhibitory charge received by hippocampal neurons (Mody and Pearce, 2004). In this way, GABA released from the synaptic cleft can activate extrasynaptic GABA receptors to persistently inhibit neurons (Li et al., 2016). Furthermore, α5 GABA_*A*_ receptor-mediated tonic inhibitory conductance in hippocampal pyramidal neurons may regulate memory and neuroexcitatory disorders (Caraiscos et al., 2004). Moreover, α5 GABA_*A*_ receptor activity predominates over synaptic inhibition in modifying the strength of both synaptic plasticity *in vitro* and certain forms of memory *in vivo* under specific conditions (Martin et al., 2010). Tonic inhibition could be deemed a continuous “brake on the system,” regulating excitation due to long-lasting hyperpolarization (Schipper et al., 2016). Therefore, tonic currents play an important role in mediating neuronal excitability, network oscillations (Mann and Mody, 2010), synaptic plasticity, neurogenesis (Ge et al., 2006; Duveau et al., 2011), neuronal development (Holter et al., 2010), information processing, and cognition (Martin et al., 2010).

Cortical microcircuits containing the hippocampus are critical for normal cognitive functions in the mammalian brain (Booker and Vida, 2018). Microcircuits comprise two major neuronal classes: excitatory principal cells and inhibitory interneurons, which release the neurotransmitters glutamate and GABA, respectively (Maccaferri and Lacaille, 2003; Booker and Vida, 2018; Huang and Paul, 2019). GABA inhibitory interneurons account for 10–20% of cortical neurons (Xu et al., 2010; Meyer et al., 2011; Moore and Wehr, 2013; Hu et al., 2014). They can regulate the activity of excitatory neurons to maintain normal neural circuitry and neural network activities (Verret et al., 2012; Palop and Mucke, 2016). According to differential neuronal molecular expression, cortical interneurons are divided into at least five types including parvalbumin (PV) neurons, neuropeptide somatostatin (SST/SOM) neurons, neuropeptide Y (NPY) neurons, vasoactive intestinal peptide (VIP) neurons, and cholecystokinin (CCK) neurons (DeFelipe et al., 2013). Of these, SST neurons account for 20–30% and PV inhibitory interneurons 40–50% of GABA inhibitory interneurons (Wonders and Anderson, 2006). Wamsley et al. classified cortical interneurons into four types that do not overlap according to their expressed molecular markers, namely PV, SST, VIP, and RELN (Reelin, non-SST) neurons (Wamsley and Fishell, 2017). Tremblinglay et al. divided cortical interneurons into PV, SST, and ionotropic serotonin receptor 5HT3α (5HT3αR) neurons, which were further divided into VIP and non-VIP neurons (Tremblay et al., 2016). Rudy et al. found that PV, SST, and 5HT3αR interneurons account for nearly 100% of neocortical GABAergic neurons, representing 40, 30, and 30%, respectively (Rudy et al., 2011). Neurogliaform cells, also called spiderweb cells, belong to VIP-negative/SST-negative 5HT3αR neurons (Rudy et al., 2011). In rat and human cerebral cortex, neurogliaform cells are unique in that they elicit slow, long-lasting, inhibitory postsynaptic potentials (IPSPs) on pyramidal cells and other interneurons through the combined activation of slow GABA_*A*_ and GABA_*B*_ receptors (Tamas et al., 2003; Olah et al., 2007). Neurogliaform cells produce hyperpolarizing responses in a large fraction of nearby neurons via axonal varicosities containing synaptic vesicles without requiring synapses to produce inhibitory responses, suggesting that the cells influence target neurons by volume release of GABA (Olah et al., 2009). Moreover, the inhibitory effect of neurogliaform cells is dependent on GABA_*A*_δ receptors, which preferentially localize to neurogliaform cells of all the cortical interneurons (Olah et al., 2009).

Early studies showed that GABA inhibitory interneurons are not vulnerable to Aβ attack (Rossor et al., 1982; Pike and Cotman, 1993). Therefore, research efforts have mostly focused on the effect of Aβ on excitatory neurons or excitatory synaptic transmission (Wang et al., 2004; Shankar et al., 2007; Li G. et al., 2009; Tackenberg and Brandt, 2009; Mucke and Selkoe, 2012). Recent studies, however, have shown that AD patients suffer from memory and cognitive impairment that is obviously different to normal age-related decline and partly due to hippocampal neuron over-activity caused by GABA inhibitory interneuron dysfunction (Huang and Mucke, 2012; Govindpani et al., 2017; Selkoe, 2019; Villette and Dutar, 2017; Table 1). These neural circuits are overactive in different transgenic AD mouse models including hAPP-J20, Tg2576, 5xFAD, 3xTg-AD, and APP/PS1 mice (Verret et al., 2012; Flanigan et al., 2014; Palop and Mucke, 2016; Shu et al., 2016; Cattaud et al., 2018; Lerdkrai et al., 2018; Table 2). The brain wave activity recorded by electroencephalography (EEG) in APP transgenic mice contains epileptiform discharges, suggesting that neural network excitability and neuronal discharge synchronization are enhanced in AD (Verret et al., 2012; Palop and Mucke, 2016; Lerdkrai et al., 2018). Since neural network activity is regulated by both excitatory and inhibitory neurons, these seemingly contradictory results suggest that Aβ not only affects the function of excitatory neurons but also inhibitory neurons, breaking down the balance between neuronal excitability and inhibition. Therefore, the role played by GABA inhibitory interneurons in AD development is attracting increasing attention.

**TABLE 1:** Alterations in GABA levels and GABAergic interneurons in AD patients.

Subtypes	Age (Years)	Subregion	Number	References
GABA level	65.73 ± 8.53	Parietal region	↓	Bai et al., 2015
GABA level	62.3 ± 6.7	CSF	↓	Bareggi et al., 1982
SST level	78–97	Frontal cortex	↓	Grouselle et al., 1998
GABA level	73.8 ± 8.4	CSF	↓	Zimmer et al., 1984

SOM(SST)–neurons	72.3 ± 2.3	Frontal and temporal lobes	↓	Beal et al., 1985, 1986
	82	Frontal and temporal lobes	↓	Candy et al., 1985
	74	Frontal and temporal lobes	↓	Davies et al., 1980; Davies and Terry, 1981

PV neurons	75–86	Neocortex	Unchanged	Ferrer et al., 1991
	81.0 ± 6.1	Neocortex	Unchanged	Hof et al., 1991
	81.2 ± 2	DG/CA1-2	↓	Brady and Mufson, 1997
	58–92	EC	↓	Solodkin et al., 1996

SOM(SST)–neurons	63–100	Olfactory tubercle and piriform cortex	↓	Mikkonen et al., 1999; Saiz-Sanchez et al., 2016
PV neurons			↓	
CR neurons		Amygdala and EC	↓	

PV neurons	86.4 ± 2.2	DG	Unchanged	Takahashi et al., 2010
CR neurons		DG	↓	

SOM(SST)	75.3 ± 13.9	Perirhinal cortex	↓	Saiz-Sanchez et al., 2013
PV neurons			↓	

GABA_*B*_R1–neurons	77.8 ± 13.9	CA1	Significantly reduced↓	Iwakiri et al., 2005
GABA_*B*_R1–immunoreactivity		CA4 and CA2/3	Increased	

**TABLE 2:** Alterations in specific subtypes of GABA interneuron in typical AD mouse models.

Model	Subtypes	Age (M)	Subregion	Number	Activity	References
hAPP-J20	PV/CR neurons	8 M	GABAergic SHP	Unchanged, but axons are decreased	↓ Hippocampal θ and γ oscillations are diminished	Rubio et al., 2012
	
	Unclassified	3 M	CA1	↓		Wright et al., 2013
			CA3	Unchanged		
	
	PV neurons	3 M/4 M–7 M	Parietal cortex	Unchanged	↓ Nav1.1 and γ oscillations are decreased	Verret et al., 2012; Martinez-Losa et al., 2018

3×Tg	PV neurons	18 M	CA1	52%↓	PV expression is decreased	Zallo et al., 2018
	CR neurons	18 M	CA1	33.7%↓	Unchanged CR expression	

APP/PS1h-o	PV neurons	10 M	CA1-2	↓		Takahashi et al., 2010
KI	CR neurons	10 M	DG and hilus	↓		

AβPPswe/PS1-dE9	PV neurons	6 M/8 M	Olfactory cortex	↓	A later and less pronounced decrease	Saiz-Sanchez et al., 2012
	CR neurons	2/4/6/8 M	Olfactory cortex	↓		
	SST neurons	4/6/8 M	Olfactory cortex	↓	Both CR and SST: an early and marked fall	

APP/PS1-KI	PV neurons	10 M	Frontal cortex	Unchanged		Lemmens et al., 2011
	CR neurons	10 M		Unchanged		

APP751_*Swed*_	PV neurons	6 M	Perirhinal cortex	Unchanged		Sanchez-Mejias et al., 2019
_*Lond*_/PS1_*M146L*_	SOM neurons			↓		

APPswe/PS1-dE9	PV neurons	3 M/12 M	CA3	↑		Verdaguer et al., 2015
	CR neurons	3 M/12 M	SGZ	↓		
	CR neurons	12 M	Hilus	↑		
	PV neurons	4 M	Hippocampus		↑	Hijazi et al., 2019

TgCRND8	NPY neurons	5,6 M	Hippocampus	↓		Ma and McLaurin, 2014, 2017
	SST neurons	5,6 M	Hippocampus	↓		

App^*NL–/NL–F*^	PV neurons	10–18 M	Dorsal LEC	↓	A reduction in the somatic inhibitory axon terminal	Petrache et al., 2019
	CR neurons	4–18 M	CA1	Unchanged		Shi et al., 2019
	CCK neurons	4–18 M	CA1	↓		
	SST neurons	9–18 M	CA1	↓		

5×FAD	PV neurons	12 M	Cortex layer IV	28.9%↓		Flanigan et al., 2014
	
	PV neurons	3 M	CA1/CA3		γ oscillations decreased↓	Iaccarino et al., 2016
		6 M	Auditory cortex CA1/mPFC		↓	Martorell et al., 2019

Tg2576	PV neurons	6 M	EC	Dendritic branch spines ↓	↓	Shu et al., 2016; Yang et al., 2018
	
	PV neurons	3 M	CA1-3		↓	Cattaud et al., 2018
	
	PV neurons	6–19 M	CA1/CA3	↓		Huh et al., 2016
Mutated *Tau* VLW line	PV neurons	2 M/8 M	GABAergic SHP	Unchanged, but axon terminals are decreased	↓	Soler et al., 2017

PS1^*M146L*^× AβPP 751^*SL*^	SOM neurons	6 M	Hippocampus	A profound ↓ diminution (50–60%)		Ramos et al., 2006
	
	CR neurons	4–12 M	CA1 and CA2/3	A substantial ↓ decrease (35–45%)		Baglietto-Vargas et al., 2010

APP695swe/PS1-dE9	PV neurons	4 M	Frontal cortex	A significant ↓ reduction (20%)		Cheng et al., 2019
	CR neurons	4 M	Frontal cortex	A decreased tendency		
Sirt3^+/–^ /APP695swe/PS1-dE9	PV and CR neurons	4 M	Frontal cortex	Significantly ↓ reduced by more than 50%		

Tau P301S and CK-p25	PV neurons	6 M	Visual cortex, prefrontal cortex, and CA1		γ oscillations are decreased ↓	Adaikkan et al., 2019

ApoE4-KI	SST neurons	6–21 M	Hilus	↓		Andrews-Zwilling et al., 2010

*↓Number: significantly decreased; Activity: significantly downregulated.*

**FIGURE 1:**
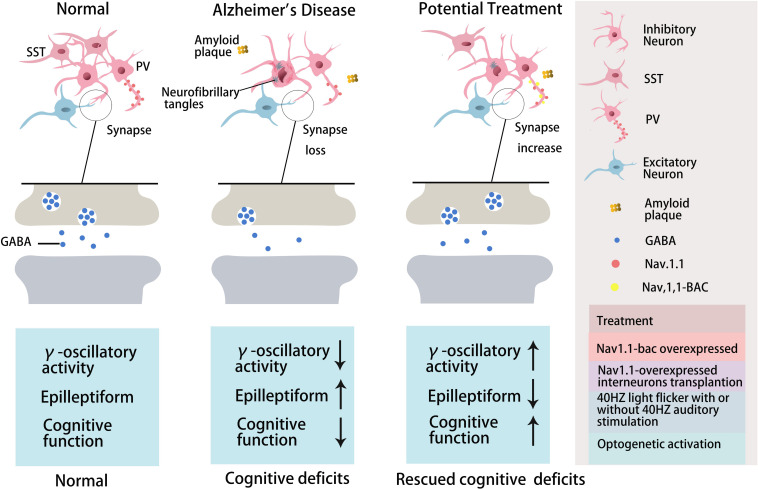
The role of GABA inhibitory interneurons, especially PV neurons and SST neurons, during AD progression and as potential treatment targets. Abnormal increased network activity in AD pathogenesis may be due to GABA inhibitory interneuron loss, synapse loss, or GABA inhibitory interneuron dysfunction, eventually leading to the development of the disease. GABA inhibitory interneurons are a potential target for AD treatment by increasing neuron number, enhancing neuronal activity, inhibiting synapses loss, or promoting GABA release. PV inhibitory interneurons in AD are dysfunctional with decreased γ-oscillatory activity, Nav1.1 expression, and GABA release (Verret et al., 2012). Improving Nav1.1 expression (Verret et al., 2012), enhancing γ-oscillatory activity in PV inhibitory interneurons by 40 Hz light flickering with/without 40 Hz auditory stimulation (Iaccarino et al., 2016; Adaikkan et al., 2019; Martorell et al., 2019), or transplanting Nav1.1-overexpressing interneurons (Verret et al., 2012; Martinez-Losa et al., 2018) could inhibit epileptiform phenomena and rescue cognitive deficits. Optogentic activation of PV and SST neurons rescued network oscillations (Chung et al., 2020; Park et al., 2020).

Further studies have indicated that abnormal Aβ production and aggregation interfere with GABA inhibitory interneuron function, aberrantly activating hippocampal neurons and promoting cognitive impairment in AD mice (Verret et al., 2012). There is abnormal hippocampal overactivity in patients with moderate AD and young people carrying the apolipoprotein E (*APOE4*) gene (Filippini et al., 2009; Yassa et al., 2010; Bakker et al., 2012). Imbalanced excitatory and inhibitory neurons in the hippocampus may be important in the cognitive impairment seen in aging and AD patients (Palop and Mucke, 2016; Li et al., 2017; Selkoe, 2019). Aβ can reduce the number and activity of GABA inhibitory interneurons, resulting in abnormal synaptic transmission and aberrant neural network activity, ultimately causing cognitive impairment in both AD patients and mice (Verret et al., 2012; Palop and Mucke, 2016; Walsh et al., 2017; Frere and Slutsky, 2018). Moreover, the altered patterns of synchronous activity might be caused by the loss of GABAergic septohippocampal pathway (SHP) axons in AD patients, which might also modulate hippocampal network activities (Rubio et al., 2012; Soler et al., 2017). Therefore, targeting GABA inhibitory interneurons to rescue cognitive impairment might be a valuable therapeutic option, perhaps by increasing the number or activity of GABA inhibitory interneurons or the number of GABA inhibitory interneuron synapses or regulating GABA neurotransmitters (Figure 1 and Table 3).

**TABLE 3:** GABA inhibitory interneurons as treatment targets in AD.

Model	Subtypes	Age (M)	Treatment	Subregion	Number	Activity	Cognitive function	References
hAPP-J20	PV neurons	3 M/4 M-7 M	Nav1.1-overexpression	Parietal cortex		↑	↑	Verret et al., 2012
	
	GABA neurons (unclassified)	7–14	Tau KO			↑	↑	Roberson et al., 2011
	
	SOM(SST)/NPY/PV	7,8 M 7,8 M	Transplantation of Nav1.1- overexpressing interneurons	Cortex and hippocampus	↑	↑	↑	Martinez-Losa et al., 2018

Tg2576	PV neurons	6 M	An enriched environment	CA1-3	↑		↑	Cattaud et al., 2018

ApoE4-KI	NPY/SST GABA neurons (unclassified)	12 M	*Tau* KO	Hilus	↑		↑	Andrews-Zwilling et al., 2010
	
		16 M	Pentobarbital	Hilus	Unchanged			
	SOM(SST)	15.5 M	Pentobarbital	Hilus	↑		↑	Tong et al., 2016
	SOM(SST)	9.5 M	Pentobarbital	Hilus				

ApoE4-KI and ApoE4-KI/hAPP-J20	SOM(SST)/ NPY/PV neurons	14 M–18 M	Transplantation	Hilus	↑	↑	↑	Tong et al., 2014

TgCRND8	SST neurons	5,6 M	α-MSH	Hippocampus	↑		↑	Ma and McLaurin, 2014, 2017

5×FAD	PV neurons	3 M	4 0 Hz light flicker	CA1/CA3		↑	↑	Iaccarino et al., 2016
	
	PV neurons	6 M	40 Hz auditory stimulation with or without light flicker	Visual cortex, prefrontal cortex and CA1		↑	↑	Martorell et al., 2019
	
	−	1–8 M	Fingolimod	Hippocampus		GABA level ↑	↑	Carreras et al., 2019

Tau P301S CK-p25	NeuN^+^ (unclassified)	8 M	40-Hz visual-stimulation	V1/CA1	↑	↑		Adaikkan et al., 2019
		6–9 M		V1/CA1/SS1/CC	↑	↑		

APP/PS1	PV neurons	4 M	Chemogenetic inhibition-CNO	Hippocampus		↓		Hijazi et al., 2019

APP/PS1	−	2 M	GABA administration	Hippocampus			↑	Sun et al., 2012

Sirt3^+/–^ /APP695swe/PS1-dE9	PV neurons CR neurons	1 M	Feeding with a ketone ester-rich diet for 24 weeks	Frontal cortex	↑ ↑			Cheng et al., 2019

APP695swe/PS1?E9	PV neurons	6 M	Injecting intraperitoneally daily for 4 weeks	Cortex	↑		Rescued impaired short-term memory	Zhang Q. et al., 2018

C57BL/6J	PV neurons	4–11 weeks	Injecting Aβ, Optogenetic manipulation	Hippocampus		↑	Rescued network oscillations	Chung et al., 2020; Park et al., 2020
PV-Cre and SST IRES-Cre mice	SST neurons					↑		

## Targeting Increased Numbers of GABA Inhibitory Interneurons

**FIGURE 2:**
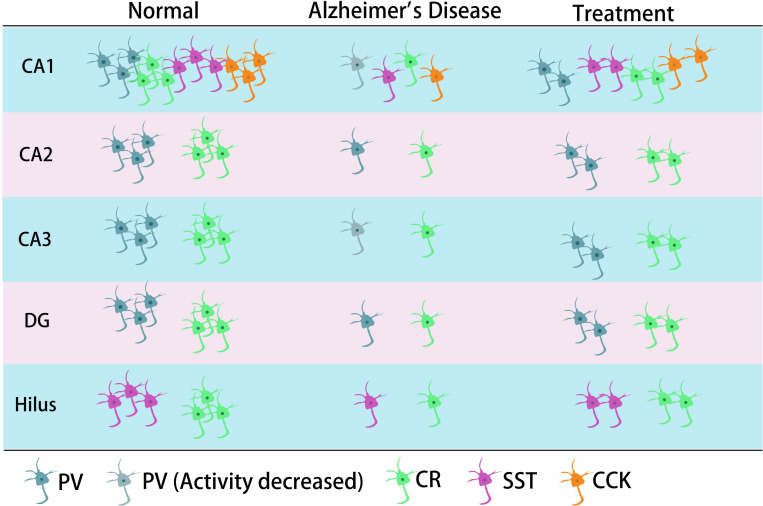
The main typical GABA inhibitory interneurons in the hippocampus involved in AD. In the CA1 region of hippocampus, it has been shown that all four types of GABA inhibitory interneuron change in number during AD. CR neurons decreased in different regions of hippocampus. The neural activity of PV neurons decreased in CA1 or/and CA3 in their corresponding studies. SST cells might also decrease in the hilus.

Previous studies have shown that neuronal loss is common in AD (Wright et al., 2013; Figures 1, 2 and Tables 1, 2). Excessive Aβ may downregulate GABA inhibitory interneuron activity, cause GABA inhibitory interneuron loss, and thereby induce functional deficiencies in GABA inhibitory interneurons (Verret et al., 2012; Palop and Mucke, 2016; Lerdkrai et al., 2018). A recent study showed that pTau accumulates in the hippocampal subgranular cell zone and hilus in AD patients. Most of these pTau-positive cells have been identified as GABAergic interneurons by co-labeling with glutamate decarboxylase 67 (GAD67) in PV and SST neurons, further suggesting GABA inhibitory interneuron function is disturbed in AD (Zheng et al., 2020). Compared with controls, GABA inhibitory interneurons and SST neurons in the brains of AD patients are abnormally reduced, along with the inhibitory neurotransmitters (Bareggi et al., 1982; Zimmer et al., 1984; Grouselle et al., 1998; Bai et al., 2015; Shetty and Bates, 2016; Table 1). There are significantly fewer SST neurons in the frontal and temporal lobes of AD patients as measured by high-performance liquid chromatography analysis or immunohistochemical staining (Davies et al., 1980; Davies and Terry, 1981; Beal et al., 1985, 1986; Candy et al., 1985). PV neurons in the temporal neocortex have been shown to be relatively resistant to degeneration in AD, with only one in seven patients with AD having significantly reduced PV neuron numbers (Ferrer et al., 1991; Hof et al., 1991). However, PV neurons are decreased in dentate gyrus (DG)-CA4 and CA1–CA2 but not in CA3 of the hippocampus in AD patients (Brady and Mufson, 1997). Furthermore, there is a clear decrease in PV neurons in parts of the entorhinal cortex in AD patients (Solodkin et al., 1996). Other studies have shown that SST neurons, calretinin (CR) neurons, and PV neurons are decreased in the olfactory tubercle, piriform cortex, amygdala, and EC of AD patients (Mikkonen et al., 1999; Saiz-Sanchez et al., 2016). Takahashi et al. observed a dramatic reduction in the mean densities of CR neurons in the hippocampal DG in AD patients compared to controls by immunofluorescent detection. However, compared to controls, there were no differences in PV neuron densities in the same region (Takahashi et al., 2010).

Later studies indicated that the numbers of GABA inhibitory interneurons in the hippocampus were significantly decreased in transgenic mice such as TgCRND8, Tg2576, 5xFAD, and TauPS2APP (Krantic et al., 2012; Loreth et al., 2012; Verret et al., 2012; Flanigan et al., 2014; Huh et al., 2016; Palop and Mucke, 2016; Shu et al., 2016; Cattaud et al., 2018; Lerdkrai et al., 2018; Carreras et al., 2019; Table 2). Loss of PV and CR interneurons in the hippocampal CA1 of aged 3xTg-AD mice might be in part due to the global network excitability defects associated with AD (Zallo et al., 2018). In APP/PS1 mice, there was a loss of PV neurons (40–50%) in CA1-2 and a loss of CR neurons (37–52%) in the DG and hilus (Brady and Mufson, 1997; Takahashi et al., 2010), as well as a loss of SST neurons (50–60%) in the hippocampus (Ramos et al., 2006). Another immunohistochemical study in this model showed a substantial decrease (35–45%) in CR-positive interneurons in CA1 and CA2/3 hippocampal subfields in very young mice (4 months) compared to age-matched controls (Baglietto-Vargas et al., 2010). At 4 months, Cheng et al. found that there was a significant reduction (20%) in PV neurons in the frontal cortex and also some decreases in CR neurons in APP/PS1 mice. SIRT3 haploinsufficiency (*Sirt3^+/–^AppPs1*) aggravated the loss of both PV and CR neurons by >50% compared to control mice and *Sirt3* haploinsufficiency alone mice (Cheng et al., 2019). Feeding with a ketone ester-rich diet increased SIRT3 expression and prevented PV and CR neuron degeneration in *Sirt3^+/–^AppPs1* AD mice, indicating that the aggravated GABAergic neuronal loss seen in these mice may be caused by reductions in SIRT3 that could be rescued by increased SIRT3 expression (Cheng et al., 2019). Further results obtained from APP/PS1 AD mice showed that there was also a decrease in PV, SST, and CR neurons in the olfactory cortex (Saiz-Sanchez et al., 2012). Moreover, CR neurons decreased during the early stages of the pathology, while PV neurons decreased at the later stages of the disease in the olfactory bulbs of APP/PS1 mice (Saiz-Sanchez et al., 2013; Zallo et al., 2018). However, there have been contradictory analyses of these neurons in AD mice, with other studies revealing no changes in either the hippocampus or the entorhinal cortex (Lemmens et al., 2011; Verdaguer et al., 2015). A recent study showed that SOM (SST)-positive neurons were significantly less frequent in the perirhinal cortex of 6-month-old APP/PS1 mice and in AD patients (91% in Braak V, VI cases) (Sanchez-Mejias et al., 2019). PV interneurons in the perirhinal cortex of APP/PS1 mice were unaffected, but a pronounced and significant loss (69%) has been detected in the corresponding area in AD patients (Sanchez-Mejias et al., 2019). Therefore, further studies are needed to ascertain the changes in the numbers of these neurons in AD models and their clinicopathological correlation with human disease (Table 2).

The synaptic imbalance seen in *App*^*NL–F/NL–F*^ AD mice results from a reduction in the number of PV interneurons and a reduction in the somatic inhibitory axon terminals in the lateral entorhinal cortex (Petrache et al., 2019). Another study indicated that CCK and SST interneurons decrease in an age-dependent manner in *App*^*NL–F/NL–F*^ AD mice (Shi et al., 2019). A novel mechanism of memory loss in AD suggests that the synaptic terminals of pyramidal neurons in EC layer II directly innervate CA1 PV neurons and are selectively degenerated in AD mice (Yang et al., 2018). Synaptic loss between the pyramidal and PV neurons disturbs the excitatory and inhibitory balance in the CA1 circuit and ultimately impairs learning and memory (Yang et al., 2018). *APOE4* is an AD susceptibility factor and high-risk gene that can significantly lower the age of onset of AD (Genin et al., 2011; Hu et al., 2015; Liu et al., 2015; Najm et al., 2019); about 60–75% of AD patients are *APOE4* gene carriers (Genin et al., 2011; Hu et al., 2015; Liu et al., 2015; Najm et al., 2019). There are GABA and SST decreases in *APOE4* carriers, resulting in higher brain activity during rest and in the face of work requiring memory (Filippini et al., 2009; Dennis et al., 2010). In *APOE* knock-in mice, the hilar GABAergic interneurons, especially SST neurons, significantly decline with age (Andrews-Zwilling et al., 2010). Enhancing GABA signaling in *APOE4* knock-in mice prevented age-dependent GABAergic interneuron decline and learning and memory deficits during middle adulthood (Tong et al., 2016). The *APPE*693Δ (Osaka) mutation is associated with familial AD, reduces the number of GABA inhibitory interneurons, and inhibits their functional activity, thereby causing a series of downstream AD symptoms (Umeda et al., 2017). The studies presented above suggest that the number of GABA inhibitory interneurons is significantly reduced during the process of cognitive deficits induced by excessive Aβ and may be accompanied by reduced neural activity. Thus, increasing numbers of GABA inhibitory interneurons might be potential therapeutic option in AD.

It has been reported that the treatment of transgenic *CRND8* mice with alpha-melanocyte stimulating hormone (MSH) can prevent cognitive deficits by inhibiting the loss of hippocampal SST inhibitory interneurons (Ma and McLaurin, 2014, 2017). Enriched environment rescues PV cell numbers and improves cognitive deficits (Cattaud et al., 2018). Citalopram, a selective serotonin reuptake inhibitor (SSRI), can reduce Aβ formation *in vitro* and reduce Aβ plaques in APP/PS1 mice (Dhami et al., 2013). Furthermore, citalopram administration decreases the quantity of newly generated Aβ in young healthy humans (Sheline et al., 2014). Clinical studies indicate that citalopram has therapeutic effects in AD patients, including rescuing non-cognitive neuropsychiatric behaviors such as depression, anxiety, irritability, and apathy (Zhang H. et al., 2018), although the benefits on agitation and cognition remain controversial (Siddique et al., 2009; Porsteinsson et al., 2014). Results from socially isolated rats suggested that citalopram improves learning and memory by promoting synaptic plasticity and hippocampal neurogenesis (Gong et al., 2017). Furthermore, chronic citalopram administration in APP/PS1 mice rescued impaired short-term memory and ameliorated non-cognitive behavioral deficits such as sociability dysfunction, depressive-like behaviors, and repetition-like behaviors. After treatment with citalopram, PV neurons increased in the cortex but not in the hippocampus of APP/PS1 mice (Zhang Q. et al., 2018). The improvements in behavior might be due to an increase in PV neurons, since PV neurons are essential for social interactions, the progression of depression (Zhang Q. et al., 2018), and PV neurons in the prefrontal cortex can affect short-term memory in mice (Murray et al., 2015; Shang et al., 2015; Kim et al., 2016). Cortical PV neurons are indispensable for short-term memory and social interactions via cortical circuit plasticity (Murray et al., 2015). PV neurons were found to be decreased in the cortex of 6-month-old APP/PS1 mice, and citalopram treatment increased the numbers of cortical PV neurons, thus rescuing behavioral performance (Zhang Q. et al., 2018).

Transplantation of GABA progenitor neurons into the hippocampus also improved the learning and memory function of Aβ-overexpressing mice (Tong et al., 2014). Recent studies have shown that medial ganglionic eminence (MGE) progenitor cells derived from day 13.5 (E13.5) mouse embryos and transplanted into the DG hilum of 14-month-old AD model mice had the same electrophysiological characteristics as endogenous normal GABA inhibitory interneurons after 90 days (Tong et al., 2014; Shetty and Bates, 2016). These neurons regulated neuronal activity in the DG region, significantly inhibiting memory impairment in AD mice and reducing anxiety-related behaviors to normal levels (Tong et al., 2014; Shetty and Bates, 2016). *APOE4*-overexpressing mice showed GABA inhibitory interneuron loss and cognitive impairment, while *APOE4* knockout significantly increased the number of GABA inhibitory interneurons and rescued cognitive deficits (Knoferle et al., 2014). In addition, cells derived from human embryonic stem cells (hESCs) or human pluripotent stem cells (hPSCs) can be differentiated into MGE progenitor cells after induction and then transplanted into the hippocampus of AD mice (Maroof et al., 2013; Nicholas et al., 2013). In these experiments, transplanted MGE progenitor cells differentiated into GABA inhibitory interneurons with normal function and significantly improved neuronal circuit dysfunction, thereby improving the cognitive ability of AD mice (Maroof et al., 2013; Nicholas et al., 2013; Southwell et al., 2014; Shetty and Bates, 2016). Therefore, GABA inhibitory interneurons are dysfunctional during AD development, and transplantation of GABA progenitor cells may improve cognition in AD mice (Table 3). In the early stages of AD, it might be possible to improve the cognitive ability of AD patients by increasing the number of normal GABA inhibitory interneurons by transplantation.

## Targets to Improve GABA Inhibitory Interneuron Activity

There are now several studies suggesting that abnormal neuronal activity is associated with the increase in Aβ seen in AD patients (Donohue et al., 2017; Evered et al., 2019; Wen et al., 2019). Synaptic inhibition and abnormally active neural network activity may coexist in both AD mice and AD patients (Selkoe, 2019), although further studies are needed to confirm this. Clusters of hyperactive neurons have been detected near amyloid plaques, and the hyperactivity is presumably due to a relative decrease in synaptic inhibition (Busche et al., 2008). Previous studies have shown that glutamate decarboxylase 65 (GAD65) protein levels are significantly reduced in AD patients and that, in these patients, the GABAergic system is severely affected (Schwab et al., 2013). GAD65 deficits may contribute to AD pathogenesis through a loss of GABAergic inhibitory activity (Schwab et al., 2013). In hAPP-J20 mice, without inhibiting the action potential, the frequency of spontaneous inhibitory postsynaptic currents (sIPSCs) significantly decreases, as recorded in the granular cells in the hippocampal DG and pyramidal neurons in cortical layers II/III (Verret et al., 2012; Palop and Mucke, 2016), suggesting that Aβ overexpression affects the activity of GABA inhibitory interneurons (Roberson et al., 2011). Subsequent studies have shown that these GABA inhibitory interneurons are dysfunctional, and GABA release is impaired in the hippocampus of hAPP-J20 AD mice, leading to the dysfunction of neural circuits and cognitive impairment during AD development (Verret et al., 2012; Villette and Dutar, 2017). A relative decrease in synaptic inhibition contributed to neuronal hyperactivity near amyloid plaques in the cerebral cortex of APP23 × PS45 mice (Busche et al., 2008), indicating a reduction in activity of GABA inhibitory interneurons. Oriens lacunosum-moleculare (O-LM) interneurons are one type of SST-positive interneuron in the hippocampus. In APP/PS1 mice, progressive axonal loss and increased turnover of dendritic spines suggested defective O-LM interneuron connectivity, resulting in O-LM interneuron dysfunction associated with memory deficits (Schmid et al., 2016). Similarly, significant GABA inhibitory interneuron functional impairment and GABA release inhibition were found in the hippocampi of *APOE4* transgenic AD mice (Li S. et al., 2009). Therefore, GABA inhibitory interneurons not only decrease in number but also in activity during excessive Aβ-induced cognitive deficits, resulting in GABA inhibitory interneuron functional impairment. However, further studies are necessary to clarify the mechanisms of functional impairment mediated by GABA inhibitory interneurons.

With respect to the molecular mechanism underpinning GABA inhibitory interneuron functional impairment, it has been shown that the amplitude of the action potential induced in PV inhibitory interneurons in the cortex of hAPP-J20 mice is smaller than that of control mice (Verret et al., 2012). This further confirms that Aβ overexpression affects GABA inhibitory interneuron activity. Recent studies have shown that Aβ may inhibit PV inhibitory interneuron activity by interacting with ErbB4, subsequently causing pathological abnormalities and cognitive impairment (Zhang et al., 2017). Further studies have shown that APP can regulate GABA inhibitory interneuron function by changing L-type Ca^2+^ channels. The number of L-type Ca^2+^ channels and Ca^2+^ current in GABA inhibitory interneurons were obviously increased after knocking out APP by enhancing the function and plasticity of GABA inhibitory interneurons, a phenomenon reversed by re-expressing APP (Yang et al., 2009). Aβ may also affect GABA inhibitory interneuron function by regulating other ion channels. For example, the deleterious voltage-gated sodium channel Nav1.1 mutation impairs telencephalic inhibitory neurons and results in Dravet Syndrome, an intractable form of childhood epilepsy (Sun et al., 2016). Nav1.1 expression is decreased in PV inhibitory interneurons of hAPP-J20 AD mice, affecting their intrinsic excitability and inhibiting γ oscillations, thereby triggering the synchronization of excitatory pyramidal neurons and increasing abnormal network activity with characteristics similar to epilepsy (Verret et al., 2012). The γ oscillation is crucial for maintaining normal learning and memory (Verret et al., 2012; Lundqvist et al., 2016; Struber et al., 2017; Martinez-Losa et al., 2018). Upregulation of Nav1.1 in PV inhibitory interneurons enhanced PV inhibitory interneuron activity, significantly increased PV inhibitory interneuron-dependent γ oscillation, inhibited the synchronization of neural network activity, and improved the cognitive ability of AD mice (Verret et al., 2012). A further study indicated that Nav1.1-overexpressing interneuron transplants derived from the embryonic MGE enhanced PV inhibitory interneuron-dependent γ oscillatory activity, reduced network hypersynchrony, and improved cognitive functions in hAPP-J20 AD mice (Martinez-Losa et al., 2018). Since γ oscillations play a vital role in cognitive activity and neural network regulation (Sohal et al., 2009; Buzsaki and Wang, 2012; Xenos et al., 2018), many studies have shown that Aβ deposition in the brain can be reduced by enhancing γ oscillations in PV inhibitory interneurons, which may improve cognition in AD mice (Verret et al., 2012; Iaccarino et al., 2016). A non-invasive light flicker stimulus obviously enhanced PV inhibitory interneuron activity, increased γ oscillations, reduced Aβ plaque deposition and pTau levels, and ultimately improved the cognition of AD mice (Iaccarino et al., 2016; Adaikkan et al., 2019). 40 Hz visual stimulation entrained γ oscillations in the visual cortex (V1), CA1, and pre-frontal cortex, reduced neuronal and synaptic loss, modified synaptic signaling and synaptic plasticity-related proteins, and rescued spatial learning and memory in both Tau P301S and CK-p25 mice (Adaikkan et al., 2019). The Aβ levels in the cortex and hippocampus as well as the number of plaques in 5xFAD mice were significantly reduced after continuous treatment with a 40 Hz auditory stimulation, thereby inhibiting the cognitive deficits (Martorell et al., 2019). Combined 40 Hz auditory stimulation with 40 Hz light flicker further induced γ oscillations in the hippocampal CA1 and auditory cortex, reducing amyloid levels and improving memory in AD models (Martorell et al., 2019). Hippocampal *in vivo* multi-electrode recordings revealed that optogenetic activation of channelrhodopsin-2 (ChR2)-expressing SST and PV interneurons in Aβ-injected mice selectively restored Aβ induced reduction of the peak power of theta and gamma oscillations, respectively, and resynchronized CA1 pyramidal cell spikes (Chung et al., 2020). Similar studies by Park et al. (2020) revealed that Aβ-induced impairments of gamma oscillogenesis and oscillation-induced timing-dependent LTP were fully restored by optogenetic activation of PV and SST interneurons, respectively. Hence, these results suggest that SST neurons and PV neurons are potential therapeutic targets for restoring hippocampal network oscillations in early AD (Table 3 and Figures 1, 2).

It has been shown that Tau reduction prevents Aβ-induced defects in axonal transport (Vossel et al., 2010, 2015). *Tau* knockout in hAPP-J20 mice can inhibit epileptic-like EEG activity and improve learning and memory (Roberson et al., 2007, 2011), perhaps due to the increased activity of GABA inhibitory interneurons (Roberson et al., 2011). Expressing mutant Tau in the entorhinal cortex also induces excitatory neuron loss, grid cell dysfunction, and spatial memory deficits (Fu et al., 2017). Further studies have shown that Tau accumulates predominantly in excitatory neurons rather than inhibitory neurons, not only early in the entorhinal cortex but also in areas later affected by AD (Fu H. et al., 2019). These studies suggest that both Aβ and Tau can affect excitatory neurons and GABA inhibitory interneurons during AD development, probably via different mechanisms, but ultimately disturbing the balance between neuronal excitation and inhibition (Lei et al., 2016; Fu Y. et al., 2019). High levels of Tau phosphorylation induce excessive metabolism of excitatory glutamatergic neurons and GABA inhibitory interneurons, disrupting the excitation-inhibition balance and resulting in abnormal neural network activity in TauP301L mice (Nilsen et al., 2013).

Recent studies demonstrated that ErbB4 protein knockdown in PV inhibitory interneurons significantly increased LTP and improved memory ability in hAPP-J20 mice without changing Aβ plaque deposition (Zhang et al., 2017). One plausible mechanism for this is that ErbB4 ablation increases PV inhibitory interneuron activity (Zhang et al., 2017; Zhang H. et al., 2018), although this requires further confirmation. By regulating GAD67 expression in GABA inhibitory interneurons, half-quantity expression of GAD67 significantly reduced Aβ deposition and improved cognitive performance in 5xFAD mice due to elevated GABA inhibitory interneuron activity (Wang et al., 2017a). Moreover, nicotine enhanced synaptic plasticity in the CA3–CA1 synapses, and GABA receptor antagonists inhibited this enhancement, suggesting that GABAergic interneurons are required for nicotine-treated adult anti-NGF mice (AD11), a comprehensive animal model of AD (Rosato-Siri et al., 2006). However, recent studies demonstrated that at the early stage of AD (16 weeks), Aβ induces hyperexcitability of hippocampal PV interneurons and contributes to neural network dysfunction and memory impairment in APP/PS1 mice (Hijazi et al., 2019). Suppressing PV interneuron hyperexcitability restored PV interneuron properties to wild-type levels, thereby reducing inhibitory inputs into pyramidal cells and rescuing memory deficits (Hijazi et al., 2019). One possible reason for the discrepancy may be due to the different ages and models of mice used in different studies. While further studies are needed to clarify this complex issue, these results suggest that targeting GABAergic interneurons might be a potential therapeutic target for AD patients.

## Targeting GABA Neurotransmitters

Both GABA and glutamate levels are significantly decreased in the temporal cortex of AD patients, suggesting deficient synaptic function and neuronal transmission in AD (Gueli and Taibi, 2013; Li et al., 2016). Using magnetic resonance spectroscopy (^1^H-MRS), a significant decrease in GABA levels was detected in the parietal region of AD patients (Bai et al., 2015). Further studies showed that the decreased GABA neurotransmitter levels in the CSF were associated with age and AD (Bareggi et al., 1982; Zimmer et al., 1984; Grouselle et al., 1998; Li et al., 2016). SST expression was reduced by 50% in AD, which was related to the formation of Aβ oligomers (Saiz-Sanchez et al., 2020). SST might inhibit Aβ metabolism by upregulating neprilysin activity, with elevated neprilysin activity reducing the accumulation of both soluble and fibrillar Aβ in APP transgenic mice (Saito et al., 2005). Recent studies revealed that SST interfered with Aβ fibrillization and promoted the formation of Aβ assemblies characterized by a 50–60 kDa core, and these findings may signify a new role for SST in AD (Wang et al., 2017b; Solarski et al., 2018). A liquid chromatography-mass spectrometry study suggested that GABA levels are significantly reduced in cultured APP/PS1 mouse hippocampal neurons and the CSF collected from the hippocampal regions in 0-, 2-, 6-, and 8-month-old APP/PS1 mice compared to age-matched controls (Sun et al., 2012). Direct administration of GABA into APP/PS1 AD mice at 2 months but not 6 or 8 months improved cognitive function, suggesting that GABA administration during early life may have potential as a treatment for AD (Sun et al., 2012). 5xFAD transgenic AD mice treated with fingolimod had restored hippocampal GABA, decreased brain Aβ levels, and inhibited activation of microglia and astrocytes, ultimately improving memory (Carreras et al., 2019). Therefore, it is possible to improve the cognitive impairment seen in AD model mice and AD patients by regulating the levels and release of GABA from GABAergic neurons during AD pathogenesis (Calvo-Flores et al., 2018; Table 4).

**TABLE 4:** GABA receptors as treatment targets in AD.

Object	Subtype	Age (M)	Treatment	Effect and mechanism	Cognitive function	Reference
Fisher/Brown Norway rat	GABA_*A*_ receptor agonist	28–31	Etazolate	Protects rat cortical neurons against Aβ-induced toxicity, stimulates sAPPα production in rat cortical neurons and in guinea pig brains, enhances the GABA_*A*_ receptor signaling, and rescues cognitive deficits.	↑	Marcade et al., 2008; Drott et al., 2010

AD patients	GABA_*A*_ receptor agonist	60–90 Years	Etazolate hy-drochloride (EHT0202)	EHT0202 is safe and well-tolerated over a 3-month treatment period. The study is of limited duration and is not powered to show efficacy. Etazolate needs to be assessed specifically in a clinical trial with a larger number of patients and over a longer treatment duration.	Not powered to show efficacy	Vellas et al., 2011; Yiannopoulou and Papageorgiou, 2013

APP-over –expressed CHO cells Tg2576	GABA_*A*_ receptor agonist	6	Baicalein	Significantly reduces the production of Aß by increasing sAPPα in APP-overexpressing CHO cells. In 6-month-old Tg2576 AD mice treated for 8 weeks, it decreases AD-like pathology together and improves cognitive performance.	↑	Zhang et al., 2013

Cultured rat cortical cells	GABA_*A*_ receptor agonist	−	Muscimol	Reduces amyloid Aβ_25__–__35_-induced neurotoxicity.		Lee et al., 2005
		12	Muscimol	Restores EEG activity and improves spatial recognition memory.	↑	Fu Y. et al., 2019
APP/PS1	antagonist	12	Bicuculline			
C57BL/6J	GABA_*A*_ receptor agonist	18	Propofol	Reduces Aβ_40_ and Aβ_42_ levels in the brain tissues of aged mice by decreasing brain levels of BACE1 for Aβ generation and increasing brain neprilysin levels to increase Aβ degradation.		Zhang et al., 2014
C57BL/6J	GABA_*A*_ receptor agonist	18	Propofol	Improves cognitive function by attenuating Aβ-induced mitochondrial dysfunction and caspase activation.	↑	Shao et al., 2014
APP/PS1		19				

Cultured rat cortical cells	GABA_*A*_ receptor agonist	−	CMZ	Provides protection against neurotoxic oligomeric Aβ1–42 by potentiating α1β2γ2 GABA_*A*_ function.		Vandevrede et al., 2014

C57BL/6J	GABA_*A*_ receptor agonist	5–8	NMZ	Reverses memory deficits induced by scopolamine.	↑	Luo et al., 2015
APP/PS1		3		Restores the CA1 LTP in hippocampal slices from APP/PS1 AD mice mediated by the α1β2γ2 GABA_*A*_ receptor.		
C57BL/6J	GABA_*A*_ receptor agonist	2–2.5	NMZ	Restores the cognition deficits induced by scopolamine.	↑	Qin et al., 2012

APP/PS1		3		Restores CA1 LTP impairment in hippocampal slices.		
APP/PS1	GABA_*A*_ receptor agonist	2.5	NMZ for 12 weeks	Restores cognition and lowers Aβ levels.	↑	Luo et al., 2016
3×Tg		10–12		Restores LTP via NO/cGMP, enhances CREB activity, reverses cognitive deficits, and reduces Aβ and pTau levels.	↑	
EFAD(APOE)		3.5		Lowers Aβ and elevates CREB phosphorylation and PSD-95 levels.	↑	
Aldh2^–/–^		3		Restores synaptic plasticity and attenuates the level of Aβ and pTau.	↑	

Wistar Rat	A maleate salt of NMZ	Postnatal 12 h	NMZM	Alleviates LTP suppression induced by scopolamine in the DG, partly dependent on the potentiation of GABA_*A*_ receptors.		Jiang et al., 2018
SD Rat	GABA_*A*_R positive allosteric modulator	18–24	ASA for 28 days	Has an anti-AD effect in aged rats with cognitive deficits by inhibiting neuronal injury and decreasing levels of Aβ_1–42_ in the hippocampus, ultimately rescuing the cognitive deficits; binds to GABA_*A*_R and improves cognitive function by reducing neuronal overexcitation.	↑	Chen et al., 2020

α5^–/–^ mice	α5 subunit of GABA_*A*_R	5–6	GABA_*A*_R a5 subunit ablation	Alters GABAergic synaptic transmission and enhances hippocampus-dependent memory and spatial learning ability	↑	Collinson et al., 2002
C57BL/6J	Inverse agonists of GABA_*A*_R α5 subunit	6–9	MRK-016	Increases LTP in hippocampal slices.		Atack et al., 2009
Lister rat				Enhanced cognition.	↑	

C57BL/6J	Inverse agonists of GABA_*A*_R α5 subunit	6–9	α5IA	Can potentiate LTP in mouse hippocampal slices.		Dawson et al., 2006
SD rat				Enhanced cognition.	↑	
Human		22 years 72 years	α5IA	Is well tolerated in young and elderly subjects and the efficacy of α5IA associated with cognitive deficits remains to be further determined.		Atack, 2010

Lister rat	Inverse agonists of GABA_*A*_R α5 subunit		α5IA-II	Improves encoding and recall but not consolidation in the Morris water maze.	↑	Collinson et al., 2006

SD rat	Agonists of α7-nAChR	5–8 weeks	FRM-17848	Enhances LTP in rat septo-hippocampal slices, at least in part dependent on increased GABAergic neurotransmission mediated by GABA_*A*_ α5-receptors.	↑	Townsend et al., 2016
APP/PS1	Inhibitor of Maob	10–12	Selegiline-approved for patients with PD by FDA	Impaired spike probability, synaptic plasticity, and learning and memory can be fully restored by inhibiting GABA production or release from reactive astrocytes.	↑	Jo et al., 2014
SD rats	GABA_*B*_ receptor antagonist	1/3/27	SGS742 (CGP36742)	Blocks the late IPSP and the PPI of population spikes recorded from CA1 pyramidal neurons of the hippocampus of rats *in vitro* and *in vivo*; enhances the release of glutamate, aspartate, glycine, and somatostatin *in vivo*; induces significant enhancement of the mRNA and protein levels of NGF and BDNF in the cortex and hippocampus of rats.	↑	Froestl et al., 2004
AD patients	GABA_*B*_ receptor antagonist	59–85 years	SGS742	Oral administration of SGS742 for 8 weeks significantly improves attention and working memory in patients with mild cognitive impairment and mild-moderate AD.	↑	
Long-evans rats	GABA_*B*_ receptor antagonist	3	SGS742	Acute *in vivo* administration of SGS742 in rats improves memory at least in part due to reduced total hippocampal CREB2 activity.	↑	Helm et al., 2005

Fischer 344 rats	GABA_*B*_ receptor antagonist	6/22	CGP55845	Shows a complete reversal of olfactory discrimination learning deficits in cognitively impaired aged Fischer 344 rats.	↑	Lasarge et al., 2009

Wistar rats	GABA_*B*_ receptor antagonist	−	CGP35348	Ameliorates the learning, memory, and cognitive impairments induced by microinjection of Aβ.	↑	Almasi et al., 2018

Previous *in vitro* and *in vivo* studies have indicated that Aβ suppresses synaptic inhibition via downregulation of GABA_*A*_ receptors (Gutierres et al., 2014; Revilla et al., 2014; Bearer et al., 2015). Therefore, upregulation of GABA_*A*_ receptors may be a useful AD treatment. GABA_*A*_ receptor agonists have been tested *in vitro* and *in vivo* and shown to have a positive effect on AD pathological changes. In the foraging/homing task, etazolate, a selective GABA_*A*_ receptor modulator, significantly improved the cognitive deficits in aged rats at 28–31 months (Drott et al., 2010). A further study indicated that etazolate can protect cortical neurons against Aβ-induced toxicity due to etazolate stimulating sAPPα production in rat cortical neurons and in guinea pig brains, enhancing GABA_*A*_ receptor signaling and rescuing the cognitive deficits (Marcade et al., 2008). In a later clinical Phase IIA study, EHT0202 (etazolate hydrochloride) was shown to be safe and generally well tolerated in a 3 month, randomized, placebo-controlled, double-blind study in AD patients (Vellas et al., 2011; Yiannopoulou and Papageorgiou, 2013). Similar results were obtained using another GABA_*A*_ receptor modulator baicalein. Zhang et al. found that activating GABA_*A*_ receptors with baicalein significantly reduced Aβ production by increasing sAPPα in wild-type APP-overexpressing CHO cells and also had an effect in 6-month-old Tg2576 AD mice treated for 8 weeks, decreasing AD-like pathology and improving cognitive performance (Zhang et al., 2013). Chronic stimulation of GABA_*A*_ receptors with their agonist muscimol reduced amyloid Aβ_25__–__35_-induced neurotoxicity in cultured rat cortical cells, and the effect was completely reversed by the GABA_*A*_ receptor antagonist bicuculine (Lee et al., 2005). A further study showed that AD mice exhibit increased spontaneous EEG delta (2–4 Hz) and decreased spontaneous EEG alpha (8–12 Hz) activity in the hippocampus and decreased Y-maze EEG theta (4–8 Hz) activity in the pre-frontal cortex. Low-dose muscimol and the GABA_*A*_ receptor antagonist bicuculine reduced EEG delta activity and increased EEG theta activity in the pre-frontal cortex and improved spatial recognition memory during Y-maze testing in APP/PS1 mice (Fu H. et al., 2019). Another GABA_*A*_ receptor agonist, propofol, is a commonly used anesthetic that has been demonstrated to reduce Aβ_40_ and Aβ_42_ levels in the brain tissues of aged (18 month) mice by decreasing brain levels of BACE1 to decrease Aβ generation and increasing brain neprilysin levels to increase Aβ degradation (Zhang et al., 2014). Moreover, propofol treatment improved cognitive function by attenuating Aβ-induced mitochondrial dysfunction and caspase activation in both aged (18 month) wide-type mice and 19 month APP/PS1 mice (Shao et al., 2014).

The clinical sedative and hypnotic chlomethiazole (CMZ) is neuroprotective and has anti-inflammatory properties in animal models due to GABA_*A*_-potentiating actions *in vitro* and sedative activity *in vivo* (Marshall et al., 2000; Luo et al., 2015). CMZ protects against neurotoxic oligomeric Aβ_1__–__42_ in primary neurons (Vandevrede et al., 2014). A CMZ analogue, 4-methyl-5-(2-(nitrooxy)ethyl) thiazol-3-ium chloride (NMZ), retains the GABA_*A*_ potentiating actions of CMZ *in vitro* and sedative activity *in vivo*. NMZ, but not CMZ, restored the LTP in hippocampal slices from APP/PS1 AD mice and also restored memory consolidation in these mice (Qin et al., 2012; Luo et al., 2015). These effects of NMZ can be antagonized by the GABA_*A*_ receptor antagonist bicuculline, an allosteric inhibitor of GABA_*A*_ receptor channel opening, indicating that NMZ’s neuroprotective effect is mediated by the GABA_*A*_ receptor (Qin et al., 2012; Luo et al., 2015). A further study revealed that the effect of NMZ is mediated by the α1β2γ2 GABA_*A*_ receptor without having direct actions on the ion current gated by this receptor (Luo et al., 2015). However, the mechanisms of action of NMZ as an anti-AD agent remains unclear. Previous studies have indicated that the NO/sGC/cGMP/CREB pathway is vital for cognition, learning and memory, and neuroprotective effects (Thatcher et al., 2004). NMZ is one of a series of NO-releasing hybrid agents that can be used as potential therapeutic compound to treat AD. A recent study showed that NMZ is a multifunctional drug that does not directly target Aβ and Tau pathology. NMZ successfully attenuated the hallmarks of AD pathology and rescued cognitive deficits in different mouse models of AD including three familial AD models (APP/PS1, 3xTg, APOE4) and a novel model of sporadic *Aldh2*^–/–^ AD mice in different ways (Luo et al., 2016). In APP/PS1 mice, NMZ restored cognition and reduced Aβ levels. In 3xTg mice, NMZ restored LTP via NO/cGMP, enhanced CREB activity, reversed cognitive deficits, and reduced Aβ and pTau levels. In *APOE4* transgenic AD mice, NMZ lowered Aβ and elevated CREB phosphorylation and PSD-95. In *Aldh2*^–/–^ mice, NMZ restored synaptic plasticity and attenuated Aβ and pTau levels (Luo et al., 2016). These multifunctional properties of NMZ make it a potential drug to treat the mixed pathology seen in AD patients. NMZM is a maleate salt of NMZ with higher efficacy due to its improved solubility and absorption. A recent study showed that NMZM may improve learning and memory by alleviating LTP suppression induced by scopolamine in rat DGs. Moreover, the protective effects of NMZM against scopolamine-induced depression of LTP was partly dependent on the potentiation of GABA_*A*_ receptors (Jiang et al., 2018).

Alpha-asarone (ASA) is an essential oil isolated from the traditional Chinese medicinal herb *Acorus gramineus* that has been used to treat respiratory diseases and neural disorders for centuries in traditional Chinese and Indian herbal medicine (Huang et al., 2013; Rajput et al., 2014; Chen et al., 2020). ASA has positive neuroprotective effects and improves cognition in rodent models (Gu et al., 2010; Kumar et al., 2012; Mao et al., 2015). Chen et al. found that ASA had an anti-AD effect in aged rats with cognitive deficits by inhibiting neuronal injury and decreasing Aβ_1–42_ levels in the hippocampus, ultimately rescuing the cognitive deficits (Chen et al., 2020). ASA also had neuroprotective effects on primary hippocampal neurons impaired by glutamate. Further computer modeling and whole-cell patch-clamp recording studies suggested that ASA, as a GABA_*A*_R-positive allosteric modulator, can bind to GABA_*A*_R and improve cognitive function by reducing neuronal overexcitation (Chen et al., 2020). Therefore, these studies suggest that treatment with GABA_*A*_ receptor agonists or positive allosteric regulators could be a potential strategy for improving cognitive function in the elderly and in AD patients.

GABA_*A*_ receptors are ligand-gated pentameric structures around a central chloride channel assembled from different combinations of 19 subunits including α1–6, β1–3,γ1–3, ρ1–3, δ, θ, ε, and π (Olsen and Sieghart, 2008; Sigel and Steinmann, 2012; Han et al., 2019). Different subunits may have specific functions. For example, the postsynaptic γ2 subunits mainly mediate phasic inhibition (Schweizer et al., 2003; Farrant and Nusser, 2005), while extrasynaptic GABA_*A*_ receptors contain π subunits that mediate tonic inhibition in most brain regions, with the α5 and δ subunits the major GABA_*A*_ receptors for mediating inhibition in the hippocampus (Glykys et al., 2008). α5-containing GABA_*A*_ receptors play an important role in cognitive processes by controlling a component of synaptic transmission in the hippocampus (Crestani et al., 2002). Ablation or reduction of the a5 subunit of GABA_*A*_ receptors alters GABAergic synaptic transmission and enhances hippocampus-dependent memory and spatial learning in mice (Collinson et al., 2002). Many studies have shown that inverse agonists of the GABA_*A*_ receptor α5 subunit negatively regulate receptor activity by binding to the α5 subunit to improve learning and memory. MRK-016 (Atack et al., 2009), α5IA (Dawson et al., 2006), and α5IA-II (Collinson et al., 2006) have shown positive effects in improving cognition in animal models (Gabriella and Giovanna, 2010). In a preclinical and clinical study, α5IA was well tolerated in young and elderly subjects, and the efficacy of α5IA with respect to cognitive deficits requires further clarification (Atack, 2010). 3-iodo-8-(pyridin-4-ylmethoxy)pyrazolo[5,1-c][1,2,4]benzotriazine 5-oxide 1 selectively, safely, and significantly improved mouse memory processes in strict chemical relationships with α5IA, α5IA-II, and MRK-016 (Guerrini et al., 2009). Several other compounds with pyrazolo[5,1-c][1,2,4]benzotriazine cores acting at GABA_*A*_ receptors have been confirmed to selectively display anti-amnesic and pro-cognitive activities *in vitro* and *in vivo* (Guerrini et al., 2013). α7-nicotinic acetylcholine receptor (α7-nAChR) agonists have entered clinical trials as pro-cognitive agents for treating schizophrenia and AD (Stoiljkovic et al., 2015; Townsend et al., 2016). FRM-17848, an α7-nAChR agonist, enhanced LTP in rat septo-hippocampal slices, at least in part dependent on increased GABAergic neurotransmission mediated by GABA_*A*_ α5-receptors (Townsend et al., 2016). Therefore, targeting α5-containing GABA_*A*_ receptors is an attractive strategy for treating disorders associated with cognitive defects such as AD.

A recent study found that astrocytes are also involved in tonic inhibition (Wu et al., 2014). In the DG of 5xFAD AD mice (6–8 months) and AD patients, compared with normal controls, the GABA content in astrocytes was significantly increased and the GABA release mediated by GABA transporter GAT3/4 obviously enhanced the tonic inhibition of hippocampal DG cells (Wu et al., 2014). Moreover, suppressing tonic inhibition either with SNAP-5114 to block astrocytic GABA release or with L-655,708 to block α5 GABA_*A*_ receptors rescued the LTP and memory deficits in 5xFAD AD mice (Wu et al., 2014). Consistent with this study, Jo et al. found that in APP/PS1 AD mice, activated astrocytes can induce excessive tonic gliotransmitter GABA secretion through B-type monoamine oxidase-B (Maob) and promote GABA release through the bestrophin 1 channel in DG (Jo et al., 2014). The released GABA decreased the spike probability of granule cells by acting on presynaptic GABA receptors (Jo et al., 2014). More importantly, astrocytic GABA and Maob were significantly upregulated in AD patients (Jo et al., 2014). However, the impaired spike probability, synaptic plasticity, and learning and memory could be fully restored by inhibiting GABA production or release from reactive astrocytes with selegiline, a selective and irreversible Maob inhibitor, which has been FDA-approved for patients with PD (Jo et al., 2014). Therefore, selective inhibition of astrocytic GABA synthesis, release, or GABA_*A*_ receptors may be a potential effective therapeutic strategy for treating memory impairment in AD.

**FIGURE 3:**
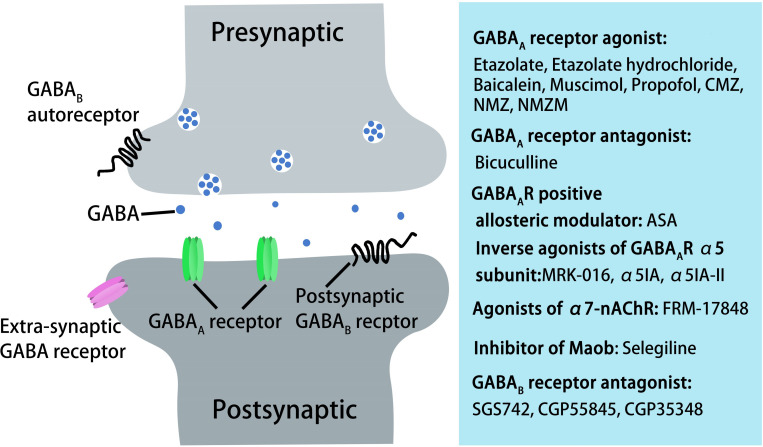
AD treatment through pharmacological manipulation of GABAergic transmission via GABA_A_ and GABA_B_ receptors. GABAA receptor agonists or antagonist, GABA_A_ receptor-positive allosteric modulators, inverse agonists of GABA_A_ receptor α5 subunit, agonists of α7-nAChR, inhibitors of Maob, or GABA_B_ receptor antagonists can rescue cognitive deficits in aged rats, AD mice, or in AD patients.

GABA_*B*_ receptors are located pre-synaptically, post-synaptically, and on extrasynaptic membranes in the hippocampus (Cryan and Kaupmann, 2005). Synaptic transmission in the brain is tightly regulated by GABA_*B*_ receptors in two ways: presynaptic GABA_*B*_ autoreceptors inhibit the release of a variety of neurotransmitters, and postsynaptic GABA_*B*_ receptors generate inhibitory K^+^ currents that hyperpolarize the membrane and inhibit neuronal activity (Cryan and Kaupmann, 2005; Dinamarca et al., 2019). Extrasynaptic GABA_*B*_ receptors can probably be activated by ‘spill-over’ of GABA from neighboring synapses (Cryan and Kaupmann, 2005). The presynaptic GABA_*B*_ receptors decline in response to neural activity (Guetg et al., 2010; Terunuma et al., 2010; Orts-Del’Immagine and Pugh, 2018) and in AD (Chu et al., 1987; Iwakiri et al., 2005). In AD patients, the numbers of GABA_*B*_ receptor R1 protein (GABA_*B*_R1)-positive neurons were found to be significantly reduced in the CA1 field of the hippocampus, and GABA_*B*_R1 immunoreactivity but not neuron number was increased in the CA4 and CA3/2 fields. The changes in hippocampal GABA_*B*_R1 may reflect the balance between excitatory and inhibitory neurotransmitter systems and result in dysfunction of the hippocampal circuitry in AD (Iwakiri et al., 2005). Recent studies have indicated that secreted Aβ precursor proteins such as sAPPα can act as GABA_*B*_R1α ligands to modulate synaptic transmission, thereby providing a potential target for the development of GABA_*B*_R signaling–specific therapeutics in AD (Rice et al., 2019). Previous reports have suggested that astrocytes are abnormally activated in AD mouse models and human AD patients (Jo et al., 2014; Wu et al., 2014). Reactive astrocytes can induce excessive tonic gliotransmitter GABA secretion, and the released GABA binds to neuronal GABA_*B*_ receptors at extrasynaptic sites, inhibiting synaptic release in APP/PS1 AD mice (Jo et al., 2014). Thus, suppressing GABA_*B*_ receptor function may rescue synaptic release and improve the cognitive deficits seen in AD. The first GABA_*B*_ receptor antagonist in clinical trials, SGS742 (CGP36742), displays pronounced cognition-enhancing effects in mice, young and old rats, and in Rhesus monkeys according to several different cognitive and learning-related behavioral tests (Getova and Bowery, 2001; Froestl et al., 2004; Helm et al., 2005). A further study has shown that SGS742 blocks the late IPSP and the paired-pulse inhibition (PPI) of population spikes recorded from rat CA1 pyramidal neurons *in vitro* and *in vivo*. SGS742 significantly enhanced the release of glutamate, aspartate, glycine, and somatostatin *in vivo*. Moreover, SGS742 induced significant enhancements in mRNA and protein levels of nerve growth factor (NGF) and brain-derived natriuretic factor (BDNF) in the cortex and hippocampus of rats (Froestl et al., 2004). Acute *in vivo* administration of SGS742 in rats improved memory, at least in part due to reduced total hippocampal CREB2 activity (Helm et al., 2005). Oral administration of SGS742 for 8 weeks in a Phase II clinical trial significantly improved attention and working memory in patients with mild cognitive impairment and mild-moderate AD (Froestl et al., 2004; Cryan and Kaupmann, 2005). Another GABA_*B*_ receptor antagonist, CGP55845, showed a complete reversal of olfactory discrimination learning deficits in cognitively impaired aged Fischer 344 rats (Lasarge et al., 2009). Further results revealed that the intra-hippocampal microinjection of the GABA_*B*_ receptor antagonist, CGP35348, ameliorated the learning, memory, and cognitive impairments induced by microinjection of Aβ in rats, suggesting that GABA_*B*_ receptor antagonism may be a therapeutic agent against the progression of acute Aβ toxicity-induced memory impairment (Almasi et al., 2018). Therefore, it is possible to improve the cognitive impairment seen in AD model mice and AD patients by regulating the level and release of GABA from GABAergic neurons and astrocytes during AD pathogenesis (Figure 3). However, the mechanisms underpinning GABA content and release remain unclear, with more research needed in this area.

## Conclusion and Future Perspectives

During AD development, Aβ leads to the loss and dysfunction of GABA inhibitory interneurons, with abnormal activity resulting in structural and functional impairment of nerve circuits and ultimately the cognitive deficits seen in AD patients and mice. Targeting GABA inhibitory interneurons by transplantation or functional enhancement may rescue the cognitive impairment seen in AD animals. Further studies of AD animal models transplanted with GABA inhibitory interneurons to stimulate GABA release or GABA receptor expression are needed. It will also be necessary to clarify the molecular mechanisms underpinning Aβ-induced dysfunction of GABA inhibitory interneurons, the aberrant activity of neuronal networks, and the cognitive deficits seen in AD. Nevertheless, GABA inhibitory interneurons represent a promising therapeutic target for the treatment of AD.

## Author Contributions

HZ and YX conceived and designed the study. YX, MZ, and YH summarized the related literature. HZ and MZ conceived the images. MZ drew the images. HZ, YX, and MZ wrote the manuscript. All authors read and approved the final manuscript.

## Conflict of Interest

The authors declare that the research was conducted in the absence of any commercial or financial relationships that could be construed as a potential conflict of interest.
